# Influence of Lignin Type on the Properties of Hemp Fiber-Reinforced Polypropylene Composites

**DOI:** 10.3390/polym16233442

**Published:** 2024-12-08

**Authors:** Florin Ciolacu, Teodor Măluțan, Gabriela Lisa, Mariana Ichim

**Affiliations:** 1 “Cristofor Simionescu” Faculty of Chemical Engineering and Environmental Protection, “Gheorghe Asachi” Technical University of Iasi, 73 Prof. Dr. Doc. D. Mangeron Blvd, 700050 Iasi, Romania; florin.ciolacu@academic.tuiasi.ro (F.C.); teodor.malutan@academic.tuiasi.ro (T.M.); gabriela.lisa@academic.tuiasi.ro (G.L.); 2 Faculty of Industrial Design and Business Management, “Gheorghe Asachi” Technical University of Iasi, 29 Prof. Dr. Doc. D. Mangeron Blvd, 700050 Iasi, Romania

**Keywords:** soda lignin, lignosulfonate, composite material, hemp reinforcement, polypropylene matrix, physico-mechanical properties, thermal analysis

## Abstract

Increasing environmental awareness has boosted interest in sustainable alternatives for binding natural reinforcing fibers in composites. Utilizing lignin, a biorenewable polymer byproduct from several industries, as a component in polymer matrices can lead to the development of more eco-friendly and high-performance composite materials. This research work aimed to investigate the effect of two types of lignin (lignosulfonate and soda lignin) on the properties of hemp fiber-reinforced polypropylene composites for furniture applications. The composites were produced by thermoforming six overlapping layers of nonwoven material. A 20% addition of soda lignin or lignosulfonate (relative to the nonwoven mass) was incorporated between the nonwoven layers made of 80% hemp and 20% polypropylene (PP). The addition of both types of lignin resulted in an increase in the tensile and bending strength of lignin-based composites, as well as a decrease in the absorbed water percentage. Compared to oriented strand board (OSB), lignin-based composites exhibited better properties. Regarding the two types of lignin used, the addition of lignosulfonate resulted in better composite properties than those containing soda lignin. Thermal analysis revealed that the thermal degradation of soda lignin begins long before the melting temperature of polypropylene. This early degradation explains the inferior properties of the composites containing soda lignin compared to those with lignosulfonate.

## 1. Introduction

Composite materials, composed of two or more constituent materials with distinct physical or chemical properties, have revolutionized modern engineering. These materials exhibit superior properties compared to their individual constituents and can be designed to meet specific performance criteria, providing custom solutions to diverse engineering problems [1,2]. To obtain composite materials, a wide range of matrices (polymers, metals, and ceramics) and reinforcements (synthetic, regenerated, and natural fibers) can be used, resulting in numerous composite types, each tailored to particular applications.

Environmental issues resulting from the depletion of nonrenewable petroleum resources and pollution have driven the research and development of sustainable composite materials with reduced environmental impact. In these materials, fossil-derived components are entirely or partially replaced by materials from renewable sources [3].

The use of natural lignocellulosic fibers as reinforcements in composite materials offers several advantages over synthetic fibers (glass, carbon, or aramid), such as renewability, biodegradability, availability, nontoxicity, low cost, and low density [4,5,6]. Among lignocellulosic fibers, hemp is one of the most resistant and stiff, providing composites that have good mechanical properties and are light weight [7]. The high strength and stiffness of hemp fibers make them suitable as reinforcing fibers in composites for structural applications that require high rigidity [8].

Over the past few years, extensive research has shown that lignin is a promising renewable and biodegradable alternative to fossil-derived materials [9].

Lignin is the second most abundant biopolymer in plant cell walls, which also contain cellulose and hemicellulose [10,11,12]. The composition of lignocellulosic biomass varies depending on the plant species (wood, crops, plants for fibers or seeds, grasses, and agricultural waste) and generally consists of 40–50% cellulose, 20–30% hemicellulose and 25–35% lignin [13,14]. Lignin, a by-product of the pulp and paper industries, has been highly underutilized so far, with only 2% being used for specialty products and the majority, 48%, being used for energy recovery [15,16]. The need to better valorize lignin has prompted the development of high value-added lignin-based products, such as adsorbents [17,18,19], dispersants [20,21,22,23,24,25], flocculants for wastewater treatment [26,27,28,29,30], supercapacitor electrodes [31,32], hydrogels for biosensor applications [33,34,35], mulch films in agriculture [36,37,38,39], bio-additives [40,41,42,43], asphalt binders [44,45,46,47,48,49,50], and controlled release fertilizers [51,52,53,54,55,56].

Lignin is not found in its pure form in nature; it is combined with cellulose and hemicelluloses. Therefore, lignin is separated either by dissolving cellulose and hemicelluloses and recovering the insoluble residue or by dissolving lignin and subsequently recovering it from the solution [57]. Depending on the method of separation, five main types of technical lignin can be obtained, each differing in composition, structure, and properties: Kraft lignin, lignosulfonate, soda lignin, organosolv lignin, and acid hydrolysis lignin [58,59]. Kraft lignin is a residue of the Kraft pulping process, where lignin is dissolved using a solution of sodium hydroxide and sodium sulfide and then precipitated by the acidification of black liquor to a pH less than 5 [60]. Kraft lignin represents about 85% of worldwide lignin production, a major part of black liquor being underutilized in the energy recovery process [61]. The sulfur content in Kraft lignin limits its further utilization. Lignosulfonate is a by-product of the sulfite pulping process which uses an aqueous solution of a sulfite or bisulfite salt of sodium, ammonium, magnesium, or calcium at a temperature of 120–150 °C [57]. Lignosulfonates contain sulfur and are characterized by water solubility, high molecular weight, and a broad molecular weight distribution [62]. Soda lignin is recovered from spent liquor in the soda pulping process, which involves treating biomass with sodium hydroxide under high-temperature and high-pressure conditions [63,64]. The soda pulping process is conducted mainly on non-wood plants (hemp, flax, grasses, and agricultural waste). Soda lignin is insoluble in water, has a low molecular weight, and contains no sulfur. Compared to Kraft lignin and lignosulfonates, soda lignin has a chemical composition that is closer to native lignin. Organosolv lignin is produced in a pulping process that uses aqueous organic solvents, such as ethanol, methanol, ethylene glycol, acetic acid, and formic acid, to treat both hardwoods and softwoods [65,66,67]. Organosolv lignin has low solubility in water, contains no sulfur, has a low molecular weight, and possesses high purity and homogeneity. However, the necessity to recover the solvents increases the cost and raises environmental concerns [15]. The acid hydrolysis process, as a direct method for separating polysaccharides and lignin from biomass by using dilute or concentrated acids, has gained increasing importance with the concept of biorefining. Acid hydrolysis lignin is characterized by a relatively lower molecular weight (2–4.5 kDa) with a dispersion index ranging from low to high values, depending on pretreatments, and a high content of methoxy groups, suitable for the synthesis of various polymers [68].

The properties, molecular structure, and reactivity of lignin are influenced by the type of feedstock used (such as hardwood, softwood, or grass biomass) and the extraction method employed for its separation. Several studies have highlighted the impact of lignin origin on the properties of composite materials [3,68,69]. Sasamori and colleagues investigated the effect of two types of lignin, one derived from pine and the other from eucalyptus, on the properties of 70/30 recycled low-density polyethylene/Pinus Elliotti wood flour composites. Their findings revealed that the mechanical properties of the composites were affected by the lignin source, with pine-derived lignin (matching the wood flour source) yielding the best performance [3]. Several researchers have also investigated the effect of the lignin extraction method on the properties of composite materials for various applications [70]. Mimini et al. reported that organosolv lignin–PLA blends exhibited better compatibility in 3D printing compared to Kraft lignin–PLA and lignosulfonate-PLA blends [71].

The aim of this research was to evaluate the potential of lignin-based composites to replace wood-based boards in furniture applications. The novelty of the current work lies in the investigation of the suitability of lignin-based composites for furniture applications by comparing their properties with those of OSB. The effect of two types of lignin (lignosulfonate and soda lignin) on the physico-mechanical and thermal properties of hemp fiber-reinforced polypropylene composites was investigated. The properties of the obtained composites were compared with those of an oriented strand board, which is traditionally used for this application. As lignin alone cannot provide the required mechanical strength and stiffness for furniture applications, a percentage of 16.65% polypropylene has been kept in the composition of the composite material. Usually, in the hemp fiber-reinforced polypropylene composites, the optimum percentage of polypropylene is 50–60% [72]. Partially replacing the PP matrix with lignin increases the biodegradability, thermal stability, and UV resistance of the composites, while also reducing environmental impact and cost.

Thermoforming has been selected as the manufacturing method for composites because it is cost-effective and provides excellent design flexibility, enabling the production of custom-shaped three-dimensional parts for upholstered furniture products [73,74].

## 2. Materials and Methods

### 2.1. Materials

A needle-punched nonwoven material, consisting of 80% hemp and 20% polypropylene, was supplied by Taparo Company (Târgu Lăpuș, Romania). The hemp fibers used to obtain the nonwoven material were cut to a length of 60 mm using a cutting machine. Hemp fibers had a tenacity of 38.9 cN/tex and a linear density of 15.8 tex. Polypropylene fibers used in the composition of the nonwoven material had the following properties: 76 mm length, 7.7 dtex linear density, 26.2 cN/tex tenacity, and 184.5% elongation at break.

A commercial soda lignin Protobind 2000 was offered by Granit Recherche Development S.A. (Lausanne, Schwitzerland). The chemical characteristics of the lignin are presented in Table 1. The average molecular weight of Protobind 2000 (soda lignin) has been reported in the literature to be approximately 5 kDa [75,76].

A commercial lignosulfonate (Lignin DS10) produced by Domsjö Fabriker AB, Örnsköldsvik, Sweden was used in this experiment.

Domsjö Lignin DS10 is a high-quality, dry powder sodium lignosulfonate product. It is manufactured in a sodium sulfite mill that produces dissolving cellulose from a feedstock consisting of a controlled mixture of softwood. The cellulose is bleached in a Totally Chlorine Free (TCF) process in a closed-loop bleaching plant (CLB).

DS10 is based on the raw material from the dissolving pulp process, which is fermented to greatly reduce the free sugars. The softwood lignosulfonate Domsjö Lignin DS10 has been described in the literature with an average molecular weight of 35–50 kDa [77,78]. The properties and analytical data of lignosulfonate are presented in Table 2.

### 2.2. Experimental Variants

To study the effect of lignin on the mechanical and thermal properties of hemp fiber-reinforced polypropylene composites, two types of lignin were used: soda lignin (Protobind 2000) and lignosulfonate. The manufactured composites and their coding are presented in Table 3.

The properties of the obtained composites were compared with those of OSB, which is traditionally used for upholstered furniture applications, along with wood and other wood-based boards. Besides the environmental benefits, replacing wood and wood-based boards with composite materials reduces the number of items and the mass of the final furniture product [79]. This is because composite materials offer exceptional flexibility in designing custom-shaped three-dimensional parts for upholstered furniture products.

### 2.3. Composite Materials Manufacturing

The nonwoven material used for composite manufacturing was produced on a technological line that includes bale openers for fiber blending, two openers for fiber tuft opening, a perforated drum for web formation, two needle-punching machines for web bonding, and a rolling device. In needle punching, barbed needles are pushed and pulled through the web to mechanically entangle the fibers, increasing the web strength through inter-fiber friction. The manufactured nonwoven fabric had a weight per unit area of 680 g/m^2^.

In the experiments, a thermal press equipped with a 40 cm × 30 cm × 20 cm mold was used to obtain composite plates.

Six pieces of nonwoven material were cut to match the dimensions of the thermopress mold and then layered. Given that the tensile strength of the needle-punched nonwoven material differs in the machine direction and the cross direction, the samples were cut both longitudinally and transversely. The layers were then alternated to ensure uniform properties.

**Figure 1:**
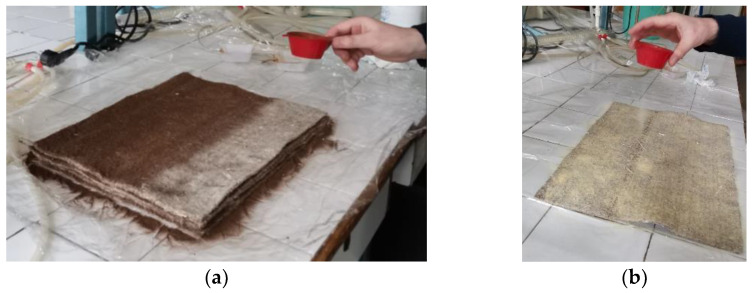
(**a**) Soda lignin sieving; (**b**) lignosulfonate sieving on the nonwoven pieces.

The nonwoven stacks were weighed, and 20% by weight of soda lignin (Protobind 2000) or lignosulfonate, respectively, was added. The lignin, in powder form, was sieved after each layer of nonwoven material, specifically between layers 1 and 2, 2 and 3, 3 and 4, 4 and 5, and 5 and 6 of the nonwoven stacks (Figure 1).

Thermoforming was performed for 15 min at a temperature of 200 °C and a pressure of 1.3 MPa, followed by a cooling period of 10 min.

### 2.4. Fourier Transform Infrared Spectroscopy

FTIR spectra were recorded using an Agilent Cary 630 FTIR spectrometer (Agilent Technologies, Santa Clara, CA, USA). The measurements were performed between 4000 and 400 cm^−1^, with 64 scans and a resolution of 4 cm^−1^, on thin samples that were sectioned from the sample with a technique of microtomy, and were then embedded in KBr.

### 2.5. Tensile and Bending Testing

**Figure 2:**
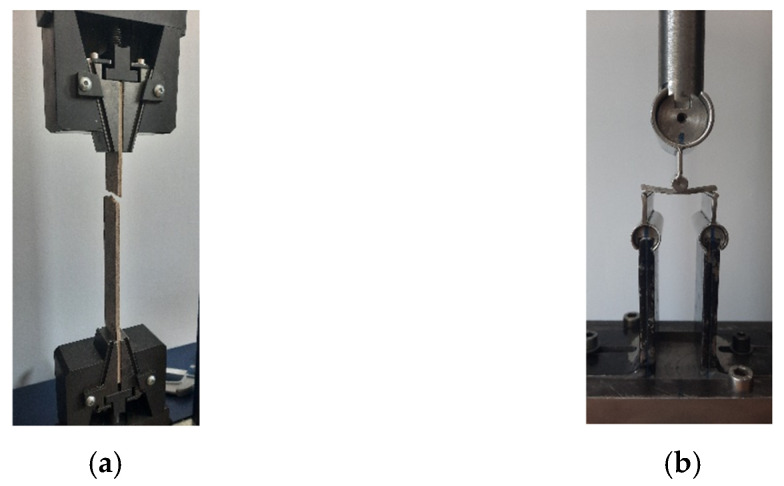
(**a**) Tensile testing; (**b**) bending testing.

Tensile tests were conducted using LBG testing equipment (Azzano San Paolo, Italy). Type 2 samples were cut to a length of 250 mm and a width of 25 mm, as specified in the ISO 527-4 standard [80]. The tensile test conditions occurred at a 150 mm distance between the grips and a test speed of 5 mm/min, in accordance with the ISO 527-4:2023 standard [81] (Figure 2a). Because the determination of the tensile strength of wood-based boards is not standardized, the same standard used for composite materials has been applied.

The 3-point bending tests for both the composite materials and the OSB were performed on the same testing machine as the tensile tests, using appropriate tools (Figure 2b). The composite material samples were 15 mm wide, with the length determined based on the sample thickness, in accordance with the ISO 14125 standard [82]. The bending tests were conducted at a cross-head speed of 2 mm/min. For the bending test of OSB, the SIST EN 310 standard [83] was applied. The sample width was 50 mm, and the length depended on the thickness of the sample. The test speed was set at 20 mm/min to ensure that breaking occurred within 60 ± 30 s.

Five repetitions were performed for each test and material.

### 2.6. Water Absorbency and Thickness Swelling

To determine the water absorbency and thickness swelling, the samples were weighed using a Kern ADJ 200-4 electronic balance (Balingen, Germany), and the thickness was measured using a caliper. Before testing, the humidity of samples was determined using a moisture balance. The samples were dipped in distilled water at room temperature. Periodically, after every 24 h, the samples were taken out of the water, wiped with absorbent paper to remove surface water, weighed, and measured.

The water absorption percentage was calculated using the following formula [84]:

(1) $$A=\frac{M_{1}-M_{0}}{M_{0}}\cdot 100\;\left(\%\right)$$

where *A* is the absorption of water, *M*_1_ is the mass of the sample after water immersion, and *M*_0_ is the mass of the sample before water immersion.

The formula used to determine the thickness swelling was as follows:

(2) $$TS=\frac{T_{1}-T_{0}}{T_{0}}\cdot 100\;\left(\%\right)$$

where *TS* is the thickness swelling, *T*_1_ is the thickness of the sample after water immersion, and *T*_0_ is the thickness of the sample before water immersion.

The measurements were repeated until the composite samples reached a constant mass and thickness.

### 2.7. Thermal Analysis

The thermal behavior evaluation of the composite materials and their components was performed using a Mettler Toledo 851^e^ device (Mettler-Toledo International Inc., Greifensee, Switzerland) and a Mettler Toledo DSC1 differential scanning calorimeter (Mettler-Toledo AG, Greifensee, Switzerland) in an inert atmosphere (nitrogen).

Thermogravimetric (TG), derivative thermogravimetric (DTG), and differential thermal (DTA) curves were recorded at a heating rate of 10 °C/min within a temperature range of 25−700 °C. The mass of the samples subjected to thermogravimetric analysis ranged between 2.25 and 5.32 mg. The nitrogen flow rate used was 20 mL/min.

DSC curves were also recorded at a heating rate of 10 °C/min in the temperature range of 25 to 230 °C, with two heating cycles and one cooling cycle. A nitrogen flow rate of 150 mL/min was used, and the mass of the recorded samples ranged between 2.92 and 5.19 mg.

The obtained TG, DTG, DTA, and DSC curves were evaluated using Mettler Toledo’s STAR^e^ SV 9.10 software. The reproducibility of the obtained results was verified by conducting multiple tests under the same operating conditions. DSC and TG tests were conducted in accordance with the standards ISO 11357-1:2023 [85] and ISO 11358-1:2022 [86], respectively.

## 3. Results and Discussions

### 3.1. FTIR Spectroscopy

**Figure 3:**
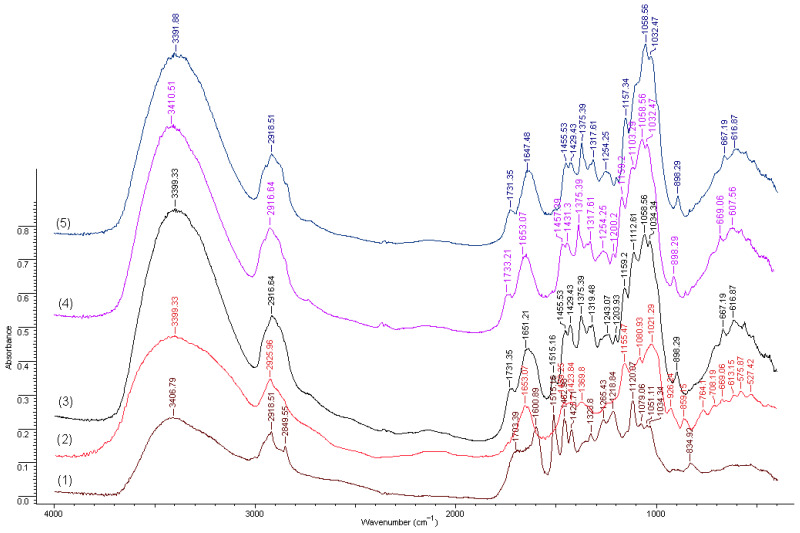
FTIR spectra of the samples: (1) Soda lignin—Protobind 2000; (2) Lignosulfonate—Domsjö Lignin DS10; (3) C-LPb2000; (4) C-LSNa; (5) C.

The FTIR spectra of the two lignins (LPb2000 and LSNa), as well as those of the composite materials obtained by adding these lignins (C, C-LPb2000, and C-LSNa), are presented in Figure 3.

The infrared spectra of the lignins are specific to their chemical structures and reflect the differences in the composition and functionality between wheat straw lignin (Protobind 2000) and softwood lignosulfonate (LSNa). Wheat straw lignin, derived from herbaceous plants, has a higher content of p-hydroxyphenyl (H) and guaiacyl (G) units, with a significantly lower content of syringyl (S) units compared to wood lignins. In contrast, softwood lignosulfonate (LSNa), derived from coniferous wood lignin, is predominantly composed of guaiacyl (G) units, with a reduced proportion of syringyl (S) units [75]. These differences are clearly reflected in the IR absorption bands specific to each type of lignin.

Both lignins exhibit common features, with bands at 1600, 1515, and 1425 cm⁻^1^ corresponding to the vibrations of the aromatic ring of the phenylpropane unit (C_9_), as well as a broad band in the wavenumber range 3500–3100 cm⁻^1^ attributed to the presence of hydroxyl groups (alcoholic and phenolic).

In terms of specific features, the bands characteristic of C-O and O-H bonds in phenols are more intense in the range 1200–1270 cm⁻^1^ and 3200–3600 cm⁻^1^ in the case of natron lignin compared to softwood lignosulfonate. Additionally, the methoxy group (-OCH₃) has weaker absorptions in the 2800–3000 cm⁻^1^ region than in lignosulfonate due to its lower content. On the other hand, the spectrum of sodium lignosulfonate from softwoods shows intense bands in the 1040–1070 cm⁻^1^ range, associated with S=O and C-S stretching vibrations due to the presence of sulfonic groups (-SO_3_⁻). The C-H bands from methoxy (-OCH_3_) are also more intense in the 2800–3000 cm⁻^1^ range, reflecting the high content of guaiacyl units.

Unfortunately, regarding the composites based on hemp and polypropylene with or without lignin additives, the FTIR spectra are not very specific since the hemp fibers themselves contain lignin in the cell wall, which prevents an individualization of the spectra through specific absorptions of the functional groups of the lignin-based additives (Pb2000, LSNa).

In conclusion, the IR spectrum of wheat straw natron lignin highlights a simpler structure with more free phenolic and aliphatic groups, while softwood lignosulfonate shows bands characteristic of sulfonic groups and a high content of guaiacyl and methoxylated units. These differences are subsequently reflected in the water affinity of the composites containing these lignin compounds.

### 3.2. Mechanical Properties

**Figure 4:**
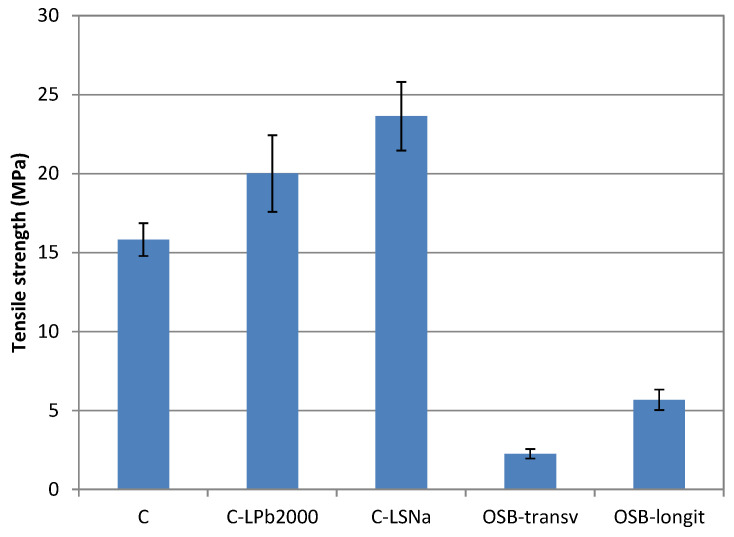
Tensile strength of lignin-based composites.

The tensile strength of the obtained composites is presented in Figure 4, along with the tensile strength of oriented strand board determined in both the longitudinal (OSB-longit) and transverse directions (OSB-transv).

When compared to the reference composite material (C), the addition of lignin resulted in an increase in the tensile strength of lignin-based composites (a 26.5% increase in the case of C-Pb2000 composite and a 49.4% increase in the case of C-LSNa composite). Lignin can act as a compatibilizer, reducing the interfacial tension between the hydrophilic natural fibers and the hydrophobic polypropylene matrix. This improved compatibility can lead to improved adhesion, more efficient stress transfer, and better mechanical properties [87]. The lignosulfonate-based composite showed a higher tensile strength than the soda lignin-based composite due to the higher molecular weight of the lignin compound, which can be attributed to both its origin (wood lignin typically has a higher molecular weight than lignin from annual plants) and the less destructive separation process. The tensile strength of all composite materials was much higher than the tensile strength of oriented strand board in both directions.

**Figure 5:**
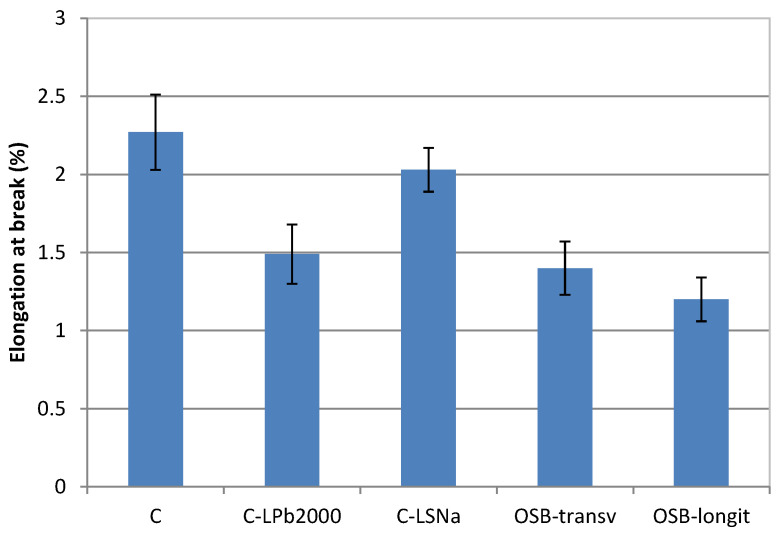
Elongation at break of lignin-based composites.

The elongation at the break of lignin-based composites is shown in Figure 5. The incorporation of lignin in the hemp/PP composite led to a reduction in the elongation at break. This is because lignin can create a more rigid structure within the composite, reducing its ductility. The addition of soda lignin reduced the elongation at break by 34.4%, while the incorporation of lignosulfonate decreased the elongation at break by 10.5%. All composite materials showed higher elongation at break than OSB. Oriented strand boards are made from thin wood strands bonded together with adhesives. Wood fibers are naturally stiffer and more brittle compared to the polymers used as matrix in composites, which leads to lower elongation at break.

**Figure 6:**
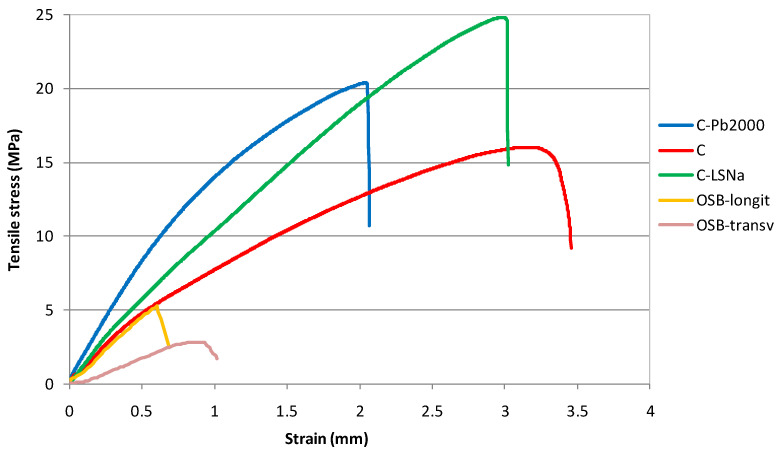
Tensile stress–strain curves of lignin-based composites.

Figure 6 shows the tensile stress–strain curves of the analyzed materials. Among the composite materials, the curve of the C-Pb2000 composite showed the steepest slope in the elastic region. This means that the material will undergo smaller strains for the same amount of applied stress compared to a composite material with a gentler slope.

Materials with a steeper slope are preferred in applications where rigidity and minimal deformation under load are critical, such as in construction materials, automotive parts, and furniture industry.

**Figure 7:**
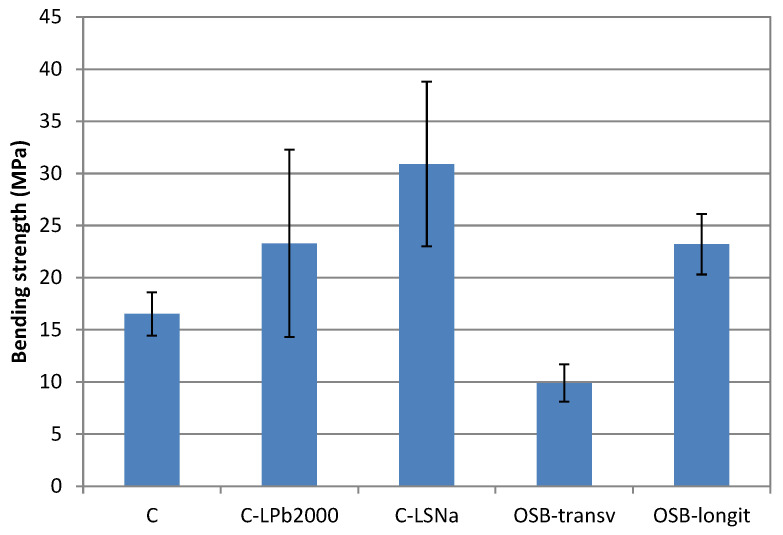
Bending strength of lignin-based composites.

Figure 7 presents the bending strength of the lignin-based composite materials. Soda lignin incorporation in the hemp/PP composite led to an increase in bending strength by 41%, while the effect of lignosulfonate addition was much more significant, causing an increase of 87%. This is because lignin is a natural stiffening agent that can enhance the rigidity of the composite material, making it less prone to bending under a given load. Also, lignin can act as a compatibilizer, improving the adhesion between the hemp fibers and the PP matrix. Enhanced adhesion results in better load transfer between composite components and higher bending strength.

When comparing the bending strength of lignin-based composites to that of OSB, it is observed that the lignosulfonate-based composite exhibited a higher bending strength than OSB in both directions. The bending strength of the soda lignin-based composite was higher than that of OSB in the transverse direction but similar in the longitudinal direction. Instead, large variability in the bending strength of the lignin-based composites was observed.

**Figure 8:**
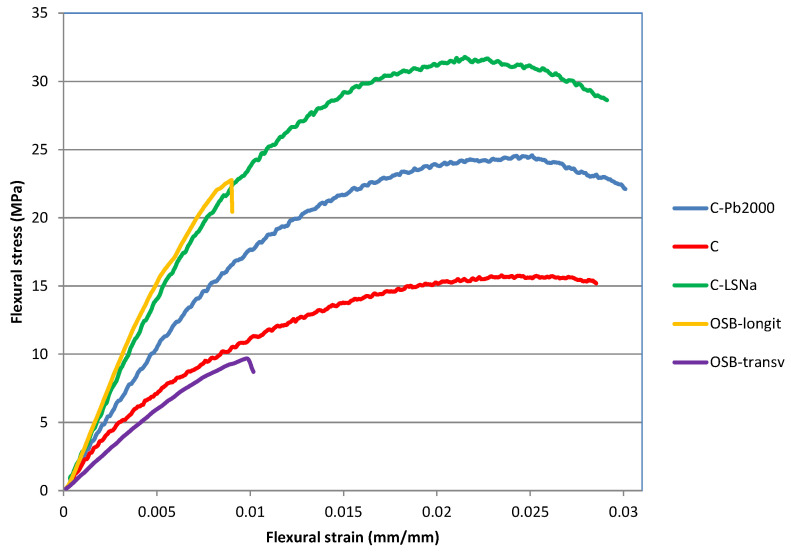
Flexural stress–strain curves of lignin-based composites.

Figure 8 presents the flexural stress–strain curves of the analyzed materials. Both lignin-based composites exhibit steeper curves compared to the C composite and OSB-transv, indicating higher stiffness and greater resistance to bending under applied loads. However, the curve of the OSB-longit sample exhibited the steepest slope, indicating the highest flexural stiffness.

### 3.3. Water Absorption Behavior

Lignocellulosic fibers are characterized by poor moisture resistance, which represents a drawback in their use as reinforcement in composites for outdoor applications in moisture-rich environments [88]. In hemp/PP composites, moisture is mainly absorbed by the hemp fibers themselves via hydrogen bonding, as well as by the interface between the hemp fibers and the PP matrix [84]. Water absorption affects the dimensional stability and the mechanical properties of composite materials.

Table 4 presents the initial thickness and density of the analyzed materials. It can be observed that lignin-based composites have a higher density than the hemp/PP composite and OSB.

**Figure 9:**
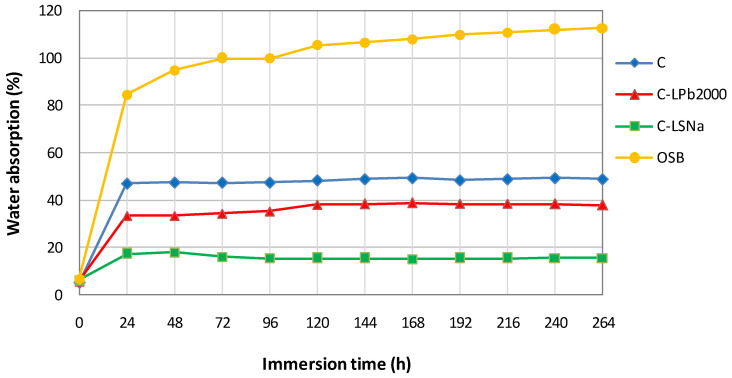
Water absorption of lignin-based composites.

The variation in water absorption over time is presented in Figure 9. The percentage of water absorption increased steeply in the first 24 h, then the increase became more gradual. It can be observed that, during the analyzed period, the OSB showed a maximum water absorption percentage of about 113%, followed by the hemp/PP composite with 49.5%. OSB is made primarily of wood strands bonded together with adhesives. Wood is a hydrophilic material that naturally absorbs water through its cellulose fibers. Composite material consists of a hydrophobic polypropylene matrix and hydrophilic hemp fibers. While hemp fibers can absorb moisture, the polypropylene matrix limits overall water absorption. Furthermore, OSB has a higher porosity than the composite material, allowing water to penetrate and be retained within the board.

Lignin-based composite materials showed a lower water absorption percentage than the hemp/PP composite due to the lower content of hemp fibers, resulting in reduced contact between water molecules and hydrophilic fibers. Moreover, lignin-based composites have a higher density and, consequently, lower porosity, which reduces water absorption.

The percentage of water absorption in the composites stabilized after 5 days.

**Figure 10:**
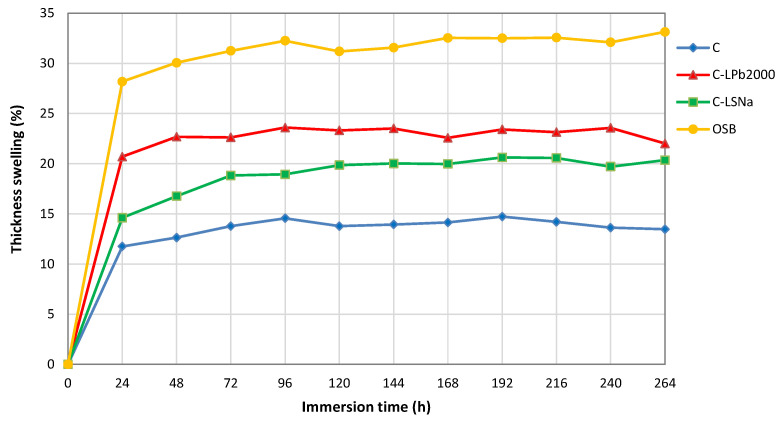
Thickness swelling of lignin-based composites.

Figure 10 presents the thickness swelling of the materials studied. The dimensions of lignocellulosic fibers change with varying moisture content. Due to the hydrophilic properties of lignocellulosic materials and capillary action, water is absorbed when the samples are soaked, leading to an increase in the dimensions of the composites [89].

As can be seen in Figure 10, all composite materials showed better dimensional stability compared to oriented strand board. The maximum thickness swelling of the composites ranged between 14.7% (C) and 23.6% (C-LPb2000), while the OSB showed a maximum thickness swelling of 32.6%.

Despite absorbing the most water, the hemp/PP composite showed the smallest thick-ness swelling. The hemp/PP composite has a lower density than the lignin-based composites (Table 4). The presence of voids within its structure enables it to absorb more water compared to the lignin-based composites. This higher water absorption is primarily attributed to the overall porosity of the composite rather than the hydrophilic nature of the fibers.

### 3.4. Thermal Properties

**Figure 11:**
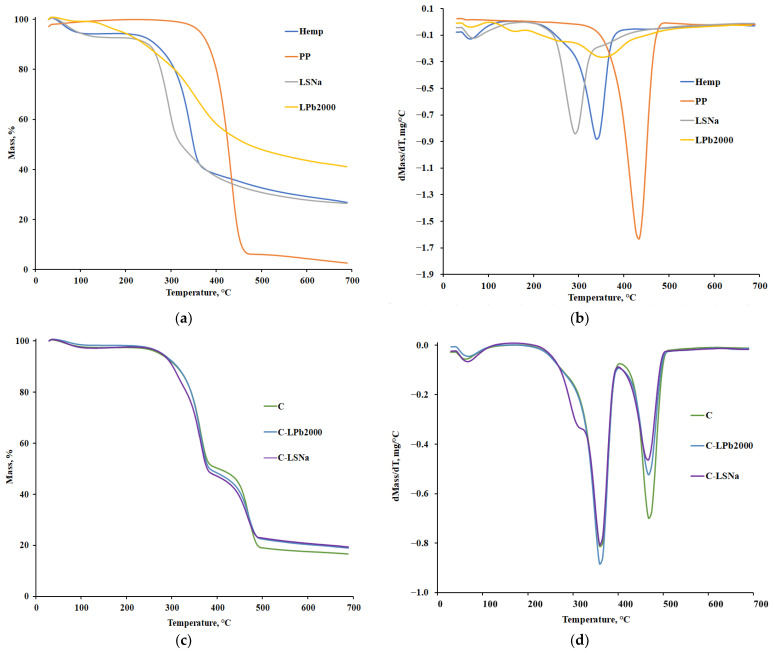
Comparative graphs of thermogravimetric analysis of components and composites: (**a**,**c**) TG curves; (**b**,**d**) DTG curves.

The thermal behavior of the components and formulated composites was investigated using thermogravimetry (TG). Figure 11a,c illustrate the curves of weight evolution versus temperature, while Figure 11b,d display the corresponding first-derivative curves.

Moreover, Table 5 presents important thermal parameters, including the degradation onset temperature (T_onset_), the maximum degradation temperature (T_max_), the temperature of degradation endset (T_endset_), and the degradation percentages at different stages of degradation during TG/DTG analysis.

It can be observed from the TG curves in Figure 11a,c that the thermal degradation of the components and formulated composites occurs in very distinct modes. The biobased components, hemp and lignin compounds, degrade in multiple stages. The first stage, which starts at lab temperature and is completed around 100 °C, is associated with moisture loss. This step can be found in thermograms for all composites reinforced with hemp and additives with lignin compounds. Besides this stage, hemp shows two other characteristic stages, one specific to the degradation of cellulose, a major chemical component representing 60−70% of the mass (Tmax = 341.5 °C), and the other is specific to the degradation of the lignin polymer, which is much more thermally stable (Tmax = 467.9 °C) [90].

The thermal decomposition of polypropylene completely occurs in an inert atmosphere within the temperature range of 380–463 °C, with the maximum degradation rate at 432 °C. This process is mainly due to the breaking of molecular chains, leading to the formation of volatile products. Obviously, the thermal degradation of lignin compounds is a more complex process that takes place through several competing reactions. During the reactions, different bond cleavages in the lignin molecule occur at a wide range of temperatures in close correlation with the bond energy [91]. The thermal degradation of lignin-based additive compounds represents a three-step process for the Protobind 2000 commercial products and a two-step process for the lignosulfonate, if the first stage of moisture removing is neglected.

According to the data presented in Table 5, which includes the main thermogravimetric data, we observe that for the C-LSNa and C-LPb2000 composites, the thermal decomposition of polypropylene begins at temperatures 67–68 °C higher, and Tmax increases by 38 °C compared to PP. This behavior can be explained by the complex and crosslinked aromatic structure of lignin, which acts as a char-forming agent, improving the thermal stability of the composite at high temperatures [92,93,94]. Thermal analysis revealed that the thermal degradation of soda lignin begins long before the melting temperature of polypropylene (T_onset_ Protobind 2000 = 130 °C vs. T_melt_ PP = 158 °C). This early degradation explains the inferior properties of the composites containing soda lignin compared to those with lignosulfonate.

In the case of the *C* composite containing polypropylene and hemp, an improvement in thermal stability at higher temperatures is also observed, a fact confirmed in other studies in the specialized literature [95,96]. The carbon resulting from the decomposition of hemp up to 380 °C acts as a barrier for the volatile decomposition products of PP and leads to an increase in its thermal stability in the composite [95].

**Figure 12:**
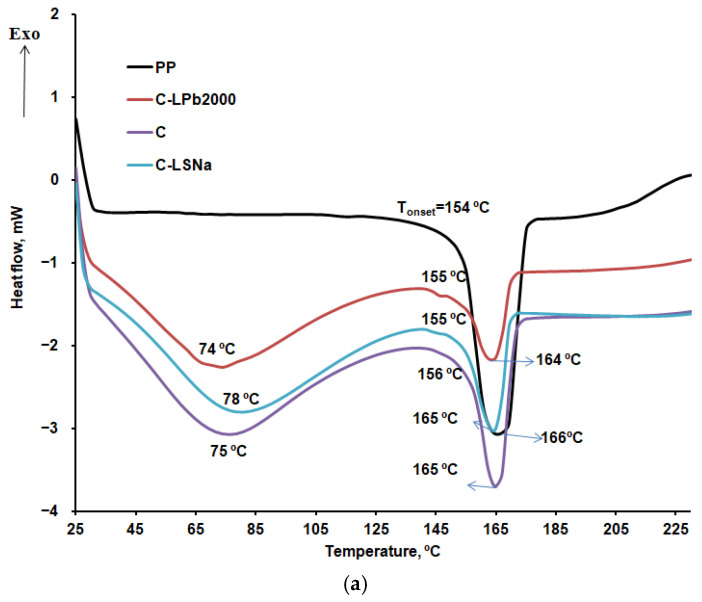

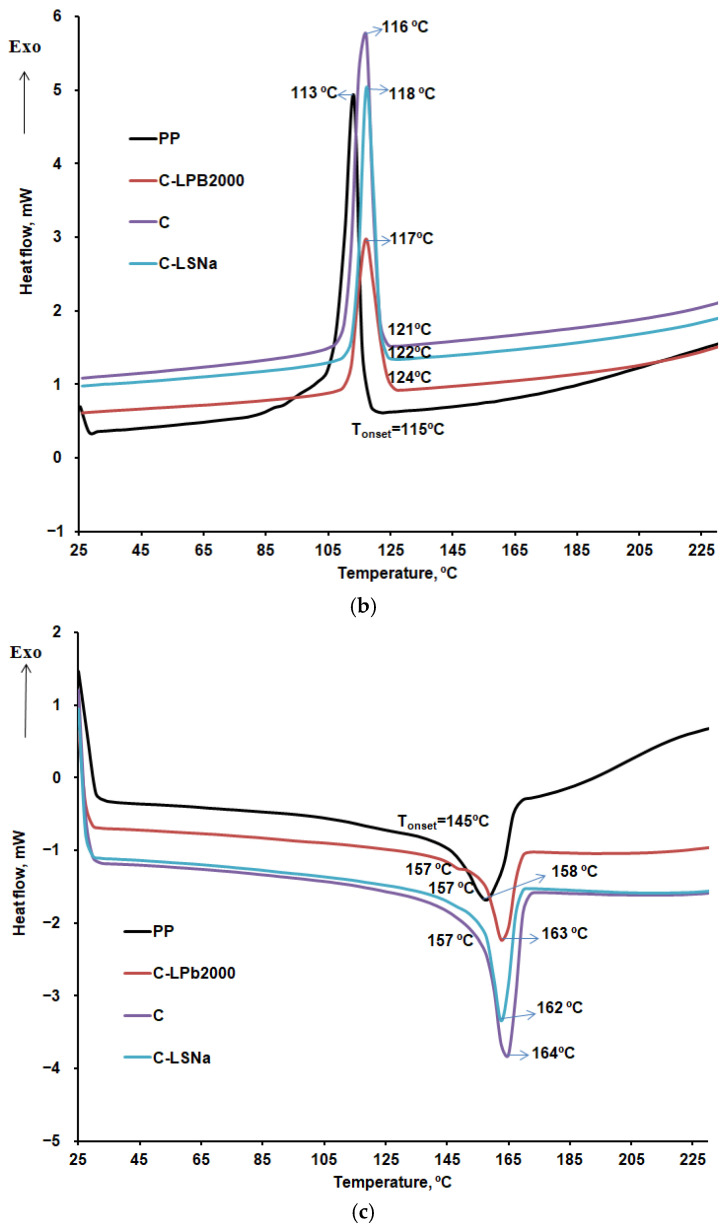
DSC curves: (**a**) first heating; (**b**) cooling; and (**c**) second heating.

The obtained DSC curves provide information about the melting temperature (Figure 12a,c) and the crystallization temperature (Figure 12b), as well as the degree of crystallinity Xc, which can be calculated using Equation (3) [97].

(3) $$X_{c}\left(\%\right)=\frac{\Delta H_{m}}{\Delta H_{m}^{\infty }}\cdot \frac{1}{f}\cdot 100$$

where $\Delta H_{m}$ is the enthalpy of melting, $\Delta H_{m}^{\infty }$ is the enthalpy of melting of a 100% crystalline PP = 207 J/g [98], and *f* is the weight fraction of the PP in the composite.

The obtained information is essential for understanding the effects of lignin and hemp fibers on the thermal transitions of polypropylene in composite materials. Table 6 presents the melting enthalpies and the degree of crystallinity, calculated using Equation (3), for PP and the composite materials during the first and second heating.

According to the obtained results, the melting temperature of the composites increases by 4 to 6 degrees compared to PP, and the crystallization temperature also increases by 3 to 5 degrees. This behavior is related to the presence of lignin and hemp fibers in the composites. The presence of hemp fibers, which can act as nucleating agents and promote the crystallization of PP, leads to an increase in the crystallization temperature and an increase in the degree of crystallinity (Table 6, sample C, which contains only hemp and PP). On the other hand, lignin is a rigid filler material that also interferes with the PP crystallization process. Due to its amorphous nature, it disrupts the orderly crystallization of the polymer matrix, resulting in a decrease in the degree of crystallinity (Table 6, samples C-LPb2000 and C-LSNa, which contain lignin, hemp, and PP). According to the Xc values presented in Table 6, a more pronounced decrease in the degree of crystallinity is observed for sample C-LPb2000, which contains soda lignin, compared to sample C-LSNa, which contains lignosulfonate. The results obtained in our study are consistent with what other researchers have reported in the literature. For example, Pengfei and others established that in PP composites containing 30% hemp, both the melting temperature and crystallization temperature increase compared to PP, and the degree of crystallinity also increases from 40% to 53% [99].

## 4. Conclusions

The research demonstrated the ability of lignin-based additives to produce composites for furniture applications with superior properties compared to both oriented strand board (OSB) and the polypropylene/hemp bicomponent composite.

The composites added with lignosulfonate have superior properties compared to those containing soda lignin, the molecular mass together with thermal stability having a decisive role. The resulting composites exhibit a balanced combination of thermal stability and crystallinity. Lignin contributes to char formation and enhances fire resistance, while hemp promotes crystallization. These properties make the composites promising eco-friendly materials with a well-rounded set of attributes, suitable for a wide range of applications.

## Figures and Tables

**Table 1:** Characteristics of Protobind 2000 lignin sample.

Characteristics	Values
Solids, %	95
Ash, %	1.3
pH (10% dispersion)	4.8
Density, g/mL	~0.6
Aromatic OH, mmole/g	1.6–1.8
COOH, mmole/g	2.1–2.3
T softening, °C	~130
Solubility in aqueous alkali, %	95

**Table 2:** Typical properties and analytical data of lignosulfonate (Lignin DS10).

Properties	Value
Dry solids, % *w*/*w*	>95%
pH (10% solution)	6 ± 1
**Analytical data**	**Typical value** **(as % *w*/*w* of total dry solids)**
Sodium, Na	9
Sulfur, S	8.5
Calcium, Ca	0.12
Chlorine, Cl	0.01
Insolubles	<0.1
Sulfate (as sulfate ions)	7.5
Total free sugars (determined by HPLC)	2.0

**Table 3:** Experimental variants.

Composite Code	Components Ratio	Composition
C	Hemp/PP = 4:1	80% hemp + 20% PP
C-LPb2000	Hemp/PP/Soda Lignin = 4:1:1	66.7% hemp + 16.65% PP + 16.65% Soda Lignin
C-LSNa	Hemp/PP/Lignosulfonate = 4:1:1	66.7% hemp + 16.65% PP + 16.65% Lignosulfonate

**Table 4:** Initial thickness and density of materials.

Material	Thickness (mm)	Density (g/cm^3^)
C	4.87 ± 0.12	0.73 ± 0.03
C-LPb2000	4.53 ± 0.15	1.10 ± 0.04
C-LSNa	4.47 ± 0.06	1.09 ± 0.02
OSB	8.1 ± 0.1	0.67 ± 0.06

**Table 5:** Characteristics of the thermal degradation process of the components and composites.

Sample	Stage	T_onset_, [°C]	T_max_, [°C]	T_endset_, [°C]	Mass Loss, [%]
Hemp	I	47.41	60.21	97.77	6.27
	II	290.67	341.59	360.09	51.14
	III	360.09	467.95	562.66	16.51
PP	I	380.39	431.69	462.29	99.99
LSNa	I	50.95	65.94	116.47	7.89
	II	258.24	292.99	310.13	38.91
	III	310.13	358.27	485.30	27.39
LPb 2000	I	50.61	59.17	75.96	1.67
	II	129.90	158.86	214.95	6.29
	III	214.95	261.50	319.18	15.17
	IV	319.18	352.10	544.83	36.82
C	I	48.49	61.72	113.07	3.00
	II	304.55	362.55	381.08	48.02
	III	446.70	469.91	488.62	32.74
C-LPb2000	I	44.84	69.33	101.86	3.63
	II	284.30	310.98	343.70	26.34
	III	343.70	361.89	378.72	28.64
	IV	449.97	470.89	486.17	25.23
C-LSNa	I	48.45	69.33	101.86	3.63
	II	284.30	310.98	343.70	26.34
	III	343.70	361.89	378.72	28.64
	IV	449.97	470.89	486.17	25.23

**Table 6:** Melting enthalpy values and degree of crystallinity for the first and second heating.

Sample	First Heating		Second Heating	
	$\Delta H_{m}$ (J/g)	X_c_ (%)	$\Delta H_{m}$	X_c_ (%)
PP	82.28	39.74	68.73	33.18
C-LPb2000	13.62	32.89	11.88	28.69
C	24.07	58.14	25.89	62.53
C-LSNa	15.94	38.50	15.09	36.44

## Data Availability

Data are contained within the article.
